# Stigmatization of Pregnant Individuals with Opioid Use Disorder

**DOI:** 10.1089/whr.2021.0112

**Published:** 2022-02-02

**Authors:** Allison D. Crawford, Kelly McGlothen-Bell, Pamela Recto, Jacqueline M. McGrath, Leticia Scott, Elizabeth A. Brownell, Lisa M. Cleveland

**Affiliations:** ^1^ The University of Texas at Austin School of Nursing, Austin, Texas, USA.; ^2^ The University of Texas Health at San Antonio School of Nursing, San Antonio, Texas, USA.

**Keywords:** stigma, pregnancy, perinatal, opioid use disorder, reproductive justice

## Abstract

***Objective:*** The purpose of this study was to critically analyze the role of stigma in the care of pregnant and parenting individuals with opioid use disorder (OUD) through the theoretical lens of the Reproductive Justice (RJ) framework.

***Background:*** Overdose related maternal mortality, often involving opioids, is a national growing public health concern. OUD is a highly stigmatized condition that may negatively influence the well-being of pregnant/parenting individual's reproductive and human rights.

***Study Design:*** Secondary qualitative data analysis.

***Methods:*** A secondary data analysis was conducted using individual interviews (*N* = 23) from a larger study aimed at examining contextual factors surrounding pregnant/parenting individual's experiences with opioid use return to use and/or overdose. The RJ framework was used as a framework to examine the influence of OUD-related stigma and a person's right to bodily autonomy, their right to parent, and their right to parent the children they have in safe and sustainable environments.

***Results:*** The RJ framework supported the examination of factors that perpetuate stigma in this population. Individuals described stigmatizing experiences in the health care setting. Verbal and nonverbal interactions with health care providers and fear of child welfare involvement were counterproductive to recovery and potentially triggered OUD recurrence and/or overdose.

***Conclusions:*** Due to existing stigma, pregnant and parenting individuals with OUD often avoided health care and recovery support services; therefore, there should be the removal of barriers that prevent this population from accessing life-saving services. Future efforts should focus on health policy-related research to support structural changes within institutions.

## Background

Overprescribing of opioids has resulted in a paralleled increase in opioid use disorder (OUD) among pregnant individuals.^1,2^ Between 1999 and 2014, prevalence rates for OUD among pregnant individuals more than quadrupled, rising from 1.5 to 6.5 per 1000 deliveries, with a substantial increase over the last decade.^3,4^ Furthermore, opioid overdose in childbearing individuals is the leading cause (58%) of death in Texas communities.^4^

OUD in pregnant and parenting individuals is a highly stigmatized health condition; expectations of this population may be incongruent with societal norms.^4,5^ Stigma develops through stereotypes, implicit bias, and discrimination enacted against a social group.^6^ As such, the stigma associated with parental OUD has historical underpinnings, associated with White supremacy and the oppression of whole populations based on class and race, and these, in turn, influence the parenting abilities of those affected by such social and structural barriers.^7^

Thus, stigma can be perceived as a global barrier to health-seeking behaviors, engagement in care, and access to recovery support services.^5^ In addition, stigma is not as well understood in the context of OUD among pregnant and parenting individuals, warranting further investigation.^6^

### Origins of stigma for pregnant and parenting individuals with OUD

The United States War-on-Drugs that began in the 1980's is followed by the current opioid crisis which has profoundly impacted U.S. health and public health practices affecting people of childbearing age at disproportionate rates.^8,9^ The changing landscape of societal regulation of laws related to substance use disorder (SUD) affecting childbearing individuals results from three pieces of federal legislation rooted in the War-on-Drugs: (1) zero tolerance policing, (2) “Just Deserts,” and (3) mandatory minimums.^4,10^ The implementation of these policies resulted in over surveillance, punitive treatment, and steep penalties for individuals with low-level crimes associated with SUD.^10^

Substance use continues to affect a large portion of the country's most marginalized people and their children.^10,11^ Moreover, since the 1980’s, the mass incarceration of this population for minor substance related charges increased over 800%,^9^ primarily impacting those who self-identify as women-of-color (60%), parents (80%), and primary caregivers (70%) to young children.^9^ Furthermore, during the federal fiscal year 2017, the rate of children entering the foster care system due to parental substance use increased for the sixth consecutive year from 2011 to 131 per 100,000 children nationally.^12^

This represents a 5% increase from the previous year and a 53% increase since fiscal year 2007. Of the nearly 270,000 children under age 18 removed from their families in fiscal year 2017, over 95,000 (36%) had “parental drug abuse” listed as the reason for their removal.^12^ These data highlight the long-lasting effects of stigma-based punitive policies on pregnant and parenting individuals with OUD and their children.

### The reproductive justice framework

Reproductive justice (RJ) represents the basic human right to maintain personal bodily autonomy, to have or not have children, and to parent children in safe and sustainable communities.^13^ For individuals with SUD, these innate rights are often violated.^12,13^ As such, we critically analyzed the impact of stigma on the reproductive rights of pregnant/parenting individuals with OUD.^13^ RJ is an ideal framework for this exploration because RJ is a combination of a Human Rights Framework and Black Feminist Theory, both of which evaluate all civil and reproductive issues.^13^

The philosophical underpinnings of RJ also acknowledge the broader social, cultural, political, and economic context that influences the development and presence of stigma by evaluating the historical nuances of the oppression which are perpetuated by societal discriminatory mindsets and institutions.^13^In this secondary analysis of an existing qualitative data set, we explore the perceptions and experiences of stigma among pregnant and parenting individuals with OUD using the three major tenets of the RJ framework as categories for characterizing stigmatizing behaviors.

## Methods

This secondary analysis (*N* = 23) included data from a larger qualitative study (*N* = 99). The data, collected throughout Texas between November 2017 and August 2018, included 13 focus groups and 23 individual interviews. Only data from the individual interviews were included in this secondary analysis because they highlight the personal stigma-related experiences of pregnant/parenting woman with OUD.

Institutional Review Board approval (HSC20160688H) and participant consent were obtained before the onset of data collection from the original study. The purpose of this study was to explore the circumstances surrounding maternal opioid-related morbidity and mortality to answer the research question: What factors contribute to parental opioid-related recurrence and overdose? The findings of this original study have been published elsewhere.^14^

### Sample

Individuals who self-identified as women were recruited from community-based, gender-specific, addiction treatment providers throughout Texas, inclusive of six geographically different regions of the state designed to ensure heterogeneous perspectives. Inclusion criteria for the study were: English speaking, at least 18 years of age, and had a “near miss” overdose or recurrence into opioid use during the perinatal period.

### Data collection and analysis

In the original study, participants were asked to describe their experience of either return to opioid use or overdose during the perinatal period. Interviews took place in a small private office space or room within a community treatment center. Probe questions were used during the individual interviews to ensure richness of data; however, participants primarily guided the discussions. Questions included: (1) Tell me about the patterns of drug use before and during your pregnancy, (2) Describe patterns of drug use the year following the birth of your baby, and (3) Can you describe events and circumstances surrounding your return to opioid use or overdose?

Interview data were transcribed verbatim by a professional transcription company, and demographic information, used to describe the sample (Table 1), was collected at the end of each interview. Participants were debriefed immediately following interviews, and all were compensated $20 for their time. Directed content analysis, guided by the RJ framework, was used to reexamine the data to allow themes to emerge with the goal of answering our research question specific to stigma.

**Table 1.** Demographics (*N*=23)

**Average age**	**34**
Ethnicity/race	
Hispanic or Latino	15
White	6
Black or African American	3
American Indian or Alaska Native	1
Relationship status	
Living with partner	7
Single	7
Married	6
Divorced	2
Widowed	1
Have three or more children	16
Highest level of education completed	
Did not complete HS/GED	11
GED/HS Diploma	6
Some college	3
Associate or higher	3
Employment	
Unemployed	18
Full-time	4
Part-time	1
Household income	
Less than $9,999	14
$10,000 to $19,000	5
$20,000 to $39,000	2
$40,000 or more	2

In addition, we sought to address trustworthiness through criticality, authenticity, integrity, and credibility.^15^ Audit trails documenting each aspect of the study and analyses enhanced criticality. We ensured authenticity using member checking; participants were asked clarifying questions following their interviews to confirm accuracy of their interview data. Integrity was accomplished through participant consent after full explanation of the study purpose, process, and procedures. Finally, all data for this secondary analysis were reviewed with the research team for confirmation of the interpretation, which ensured credibility.^15^ Collectively, this body of work contributes greatly to our understanding of the experiences of stigma for pregnant individuals with OUD.

## Results

Guided by the three basic tenets of the RJ framework, we used exemplars from our data to describe and illustrate the impact of stigma on pregnant and parenting individuals with OUD (Table 2). We confirmed that the RJ framework was ideal for this secondary data analysis since every individual represented by our study sample reported some type of stigma; most reporting examples aligned with all three of the RJ tenets.

**Table 2.** The Tenets of Reproductive Justice and their Definition

**Tenet**	**Definition**
Tenet 1	The right to bodily autonomy
Tenet 2	The right to have or not have children
Tenet 3	The right to live in safe and sustainable environments

### Tenet 1: the right to bodily autonomy

Existing evidence indicates that the right to bodily autonomy is often violated for pregnant and parenting individuals with OUD. Individuals with children experience greater stigmatization than others who use substances as this is contrary to society's beliefs of expected normative behavior in parents or those who identify as women and mothers.^4,16^

For example, the society in the United States views individuals with OUD to deviate from normative behaviors in the context of gender, expectations of pregnancy, and parenthood.^4,16^ Existing stereotypes of pregnant individuals with OUD label this population as self-destructive, “bad parents” or unfit, immoral or deficient caregivers, and even criminals.^4,5,16^ These descriptions serve as the basis for social exclusion and result in discrimination, loss of bodily autonomy and parental rights, and social isolation.^17,18^

Health care providers who hold more negative beliefs about OUD may unwittingly isolate and traumatize this population with harsh words. Rather than receiving support, some of the participants from our study described facing judgment that preceded the loss of bodily autonomy. One participant described how they perceived her doctor as ill-informed about evidence-based care specific to individuals with OUD. They described how they were denied the right to breastfeed their infant. This lack of support during the perinatal period led to them returning to opioids:

*My doctor that delivered the baby, she didn't know anything about Suboxone, and she was very iffy. And the doctor that was giving the Suboxone didn't know anything about pregnancy either. They really should have put me on the Subutex that doesn't have the naloxone in it. And they didn't know to do that, so when I had the child, the Fentanyl that they gave me didn't work because I couldn't get off the Suboxone beforehand. I was also very upset because the doctor told me I couldn't breastfeed. I just felt like no one knew what they were doing.*

Many participants in the study recounted their experiences of being excluded from participating in the care of their infants and the judgmental language they witnessed from health care providers.^14^ One individual described how they were treated in the hospital after nurses discovered they had OUD. They described being denied the opportunity to engage in the care of their child due to the existing stigma:

*I noticed that when all the nurses knew that I was on drugs at one point, they were really crappy to me. My baby was in the NICU for something totally unrelated [to prenatal opioid exposure]. I walked in there to see her and one of the nurses was feeding her. She acted like she didn't want to give me my baby.*

In addition, individuals with OUD are often disproportionately affected by profiling and punitive policies, which are deeply entrenched in our everyday systems.^19^ These people are further viewed as violators of socially defined roles as nurturing caregivers because of their substance use.^4^ As such, individuals with OUD have historically been targets of dubious birth control initiatives; the denial of evidence-based health care; pressured to have unnecessary medical procedures; and the inability to access desired health related resources.^13,20^ One participant described being denied social support during their cesarean birth due to the stigma they encountered from health care workers:

*They would move the ultrasound. I couldn't see the baby. I would ask: ‘How is she?’ The doctors wouldn't listen to me. When they were prepping me, I asked if my husband was going to go in with me into the operating room. They said: ‘Oh, hell no!’ He couldn't be in there with me. I had the baby all by myself.*

### Tenet 2: the right to have or not have children

Pregnant individuals express the challenges of overcoming stigma as they are often ostracized, criticized, and shamed.^21,22^ For pregnant individuals with OUD, self-stigma can occur when they internalize the stereotypes and prejudices assigned to them by broader society.^23^ Cultural attitudes blame people with substance use for their own morbidity and mortality and how this cultural norm fails to address the social determinants of health which only further perpetuates adverse outcomes in those with OUD.^24^

Negative labels such as “unfit” or “bad parent” for pregnant and parenting individuals with OUD may result in family separation, which has great bearing on the person's ability to have or not have children.^25^ Several participants described how the stigma of OUD influenced them during the perinatal period and how they became targeted for having all of their children removed by Child Protective Services (CPS) or child welfare. One participant described:

*CPS is snatching our kids away. That's traumatic on the kids. [CPS says] “Oh, well kids are resilient.” It's still not okay. Don't keep pushing that because these children can't take it. Some of them might grow up and have post traumatic stress disorder*.

Pregnant individuals with OUD often expressed worry over CPS involvement, which they viewed as being counterproductive to recovery. Participants described how the constant worry of child removal triggered return to use of opioids. Many were fearful of the power the child welfare system had to terminate their parental rights. As a result, participants often felt they had little input regarding their child welfare case plan and frequently did not question their plan for fear of angering individuals in positions of authority and causing retaliation. One person described how they did “everything right” according to her CPS case; however, the stigma associated with OUD still created conflict between her and the criminal justice system related to the care of their children:

*I did everything [CPS] asked me to do and [the judge] stood there on the day of our trial and said, “You show me you're not going to recover because you've had so many relapses.” Well, I'm a heroin addict, give me an option, you know? Well, the only option they gave me was to [have all my kids taken away]. They took custody of all three of my kids.*

### Tenet 3: the right to parent in safe and sustainable environments

Historically, the nation's reform and punishment systems rest on concepts of corporal punishment, public humiliation, religious penalties, financial sanctions, and forced servitude to pay off one's debt.^24^ Consequently, these concepts perpetuate the health, legal, and child welfare system, to enforce punitive measures against individuals who use substances.^26^ One person described how parents with OUD get automatically judged from society, which limited their environments of social support:

*I think that's where we fall short on not only our support, but out there in the community because we're automatically going to be judged. And that's the first thing, and that's the wrong thing. Only one person is supposed to judge, not [our neighbors].*

The participants repeatedly described how the punitive mindsets of broader society prevented them from supporting themselves and their children. They described that having limited job opportunities due to the presence of criminal records associated with their OUD. The stigma and labeling from prior convictions led them to resort to illegal activity for means of survival. One person described the challenge of securing legitimate employment and returning to illegal activities to provide for themself and their child:

*It's hard. They say they hire felons. I went to [an employer] for an interview. The manager said, “I can't wait to work with you, come for training.” And then they send me a letter in an e-mail saying, “sorry, we can't hire you because of your background.” Even though it was seven years ago, they will not hire you because of your background. I didn't wanna be doing that [illegal] stuff.*

Social judgment, blame, and stereotypes that individuals with OUD encounter are inherently dangerous and serve to perpetuate stigma.^23^ Several of the participants described judgment as a recurring theme during their interactions with the health care, CPS, and judicial system. As such, the perceived judgment interfered with the parent's attempts to interact with and raise their children in an environment that felt safe and free from stigma. One participant described how a judge involved in their child welfare case stigmatized them because of their OUD and poverty. They believed that their children were taken away from them because they were poor. They described how the loss of their children eventually led to their relapse:

*[The judge] basically, after I did all of my services, he told me that I wouldn't be able to support my children financially and he didn't believe in recovery, so he took my children, and eventually I relapsed.*

## Discussion

In this secondary data analysis, we used the RJ framework to explore the stigma surrounding OUD in pregnant and parenting individuals. The perversive and continued negative perceptions regarding pregnant and parenting individuals with OUD persist as these people often encounter stigma in the form of punitive and exclusionary practices.^27^ Many of the participants in our study recounted their experiences of being excluded from participating in the care of their infants and judgmental verbal and nonverbal language received from health care providers, all of which endanger the right to bodily autonomy.^14^

Participants in our study also reported the challenges of overcoming stigma as they internalized the stereotypes and prejudices assigned to them.

Agreement with and adoption of negative labels that they were “unfit” or “bad parents” may result in a “why try?” mentality.^25^ Feeling defeated and discouraged, individuals may altogether cease to interact with or spend time with their infants and forgo efforts that facilitate their recovery.^5^ Negative practices reinforce stereotypes and prejudice toward individuals with various health conditions, and they also foster discriminatory attitudes that fuel social inequalities.^28^ These may be embedded within communities and organizations that inadvertently reinforce stigma, thereby restricting opportunities for pregnant and parenting individuals with OUD.^27^

In the health care setting, individuals with OUD frequently state how powerless they felt against the discriminatory behaviors enacted against them.^29^ Health care providers with greater negative beliefs and attitudes about OUD may unwittingly isolate and traumatize individuals with their harsh words. Rather than receiving support, education, and encouragement, some of the participants from our study described facing judgment and condemnation. This was further detailed in their interactions with the child welfare system. The participants in our study often shared their fears of child welfare involvement, child removal, and termination of parental rights. These experiences have lasting implications on a person's right to have (or not have) subsequent children.

Negative experiences with health and judicial systems can have long-term implications on both parental and infant mental health and recovery and largely affect an individual's right to parent their child in a safe and supportive environment, free of stigma. Existing research suggests that children may play an important role in recovery.^30^ The participants in our study shared their fears of Child Welfare involvement, child removal, and termination of parental rights. Their apprehension and fears are supported by national trends.^9^

After more than a decade of steady decline, between 2012 and 2016, the United States experienced a 10% increase in children entering the foster care system.^31^ In the six states most impacted by the opioid epidemic,^32^ there was a 50% increase over this same four-year period.^31^ Design and application of innovative ideas and approaches to family preservation that prevent the multigenerational cycle of trauma that can result from parent–child separation are critical.

### Implications for research, intervention development, and policy on stigma

RJ can be helpful in formulating plans to dismantle stigma that may influence health outcomes in pregnant and parenting individuals with OUD. We will now discuss ways to promote public awareness about the negative impact of stigma, advocate for health policy change, and call for an end to the marginalization of this population of individuals. We call awareness to the misconception that OUD is a criminal matter and recommend the development of policies aligned with the science that clearly states, “Addiction is a treatable, chronic, medical disease involving complex interactions among brain circuits, genetics, the environment, and an individual's life experiences.”^33^

Despite current research, punitive policies are still enacted today in our legal systems.^9^ The American Medical Association Opioid Task Force (2020)^34^ and the American Academy of Nursing^35^ have put forth an urgent call to action to end the threat of incarceration and punitive civil actions against individuals based solely upon their history of substance use. Nurses are often at the frontlines, taking an active role in care coordination for this population. Therefore, it is critical that as health care leaders, nurses take an active stand against the harmful stigma that can jeopardize the health of parents and children.

For example, while there is no universally agreed upon treatment protocol for infants with prenatal opioid exposure, care is typically provided in a neonatal intensive care unit (NICU) where infants may experience a long hospital stay.^36^ Symptom management usually begins with common soothing techniques used for any fussy baby, including swaddling, rocking, skin-to-skin holding, and breastfeeding.

However, due to the nature of most NICUs, infants are often separated from their mothers, which is a barrier to these recommended soothing techniques. Furthermore, if the hospital environment is such that the mother is unable to be present or feels uncomfortable visiting her infant, both the mother's capacity to engage in the care of her infant, as well as her ability to sooth and comfort her own infant, may be jeopardized.

Experts are evaluating more closely whether the NICU is the most supportive and cost-effective environment for infants with neonatal abstinence syndrome (NAS).^37^ One alternative approach has been coined “Eat, Sleep, and Console (ESC)” which is a practical individualized model of care that can be implemented by nurses.^38^ ESC actively engages the biological parent in their infant's care and calls for pharmacologic intervention only when needed rather than basing medication decisions on a complicated scoring system and treatment protocol.^39^ Hospitals using the ESC model have benefited from shorter, less costly hospital stays for infants with NAS.^38,40,41^ These findings further suggest that the ESC model may be a more humane and less stigmatizing approach to caring for infants with NAS.

Additional considerations include the unique gendered needs of individuals with OUD.^42^ For example, two-thirds of individuals who self-identify as women with OUD are the primary caregivers of young dependent children.^43^ Yet, treatment and recovery support services are often not designed with the needs of parenting individuals in mind.^9^ Furthermore, some methadone clinics prohibit their clients from bringing anyone with them, to include dependent children, during daily dosing. This can serve as a significant barrier for parenting individuals in need of evidence-based treatment who have no assistance with childcare. Furthermore, few recovery residences offer beds for parents and children.^44^

Emerging literature suggests that when children are present in recovery housing, all residents benefit.^45^ Furthermore, when parents remain with their children while accessing treatment and recovery support services, they are more likely to complete treatment and enter long-term recovery.^45^ Future care models must be more gender tailored to include the unique aspects of parenthood. Several examples of such programs include: Maternal Opiate Medical Supports Plus (MOMS+) Ohio program,^46^ the Mommies Program of Texas,^47^ and Casa Mia in San Antonio, Texas.^48^

As a final consideration, while the majority of participants in our study self-reported as women, Hispanic/Latino, participants did not mention race and ethnicity as a dimension specifically shaping their experiences of stigma related to OUD. Nonetheless, evidence suggests that the right to parent has been neglected for individuals of color in the United States.^13^ Specifically, to be a parent of color with OUD comes with a set of social assumptions related to race that may also intersect with stigma associated with other statuses of oppressive positionality.^13^ Therefore, future studies should examine how the stigma surrounding OUD may intersect with other existing biases related to race, gender, sexuality, class, and the positionality of being a parent.^13^

## Conclusion

Using the RJ framework, we described the reproductive and civil injustices which propagate stigma in pregnant and parenting individuals with OUD in the context of existing evidence. To develop effective programs and interventions that alleviate the effects of stigma, it is critical to understand the systemic barriers that perpetuate it and ultimately disempowers pregnant/parenting individuals with OUD.

We illustrated the impact of stigma on pregnant individuals with OUD. By highlighting key areas for intervention development, these collective research findings should fundamentally influence health care, the delivery of social services, and public health policy reform. Greater efforts need to be directed toward developing programs targeting the needs of this unique population, and, in turn, enhance recovery.

The topic of substance use in pregnant/parenting individuals is a social issue that requires compassion, empathy, and evidence-based interventions to combat stigma. Health care providers and criminal justice/child welfare professionals who interface with these individuals play a critical role in dismantling health-related stigma with the end goal of empowerment and reproductive bodily autonomy. Due to existing stigma, pregnant and parenting individuals with OUD often avoided health care and recovery support services; therefore, there should be the removal of barriers that prevent this population from accessing life-saving services. Future efforts should focus on health policy-related research to support structural changes within institutions.

## Copyright Release

We release the copyright for our article.

## Abbreviations Used

CPS: Child Protective Services
ESC: Eat, Sleep, and Console
MOMS+: Maternal Opiate Medical Supports Plus
NAS: neonatal abstinence syndrome
NICU: neonatal intensive care unit
OUD: opioid use disorder
RJ: Reproductive Justice
SUD: substance use disorder
